# Microwave Cooking Enriches the Nanoscale and Short/Long-Range Orders of the Resulting *indica* Rice Starch Undergoing Storage

**DOI:** 10.3390/foods11040501

**Published:** 2022-02-10

**Authors:** Qing Xiong, Dongling Qiao, Meng Niu, Yan Xu, Caihua Jia, Siming Zhao, Nannan Li, Binjia Zhang

**Affiliations:** 1 Group for Cereals and Oils Processing, College of Food Science and Technology, Key Laboratory of Environment Correlative Dietology (Ministry of Education), Huazhong Agricultural University, Wuhan 430070, China; xiongqing118@gmail.com (Q.X.); nmjay@mail.hzau.edu.cn (M.N.); xuyan@mail.hzau.edu.cn (Y.X.); chjia@mail.hzau.edu.cn (C.J.); zsmjx@mail.hzau.ed.cn (S.Z.); 2 Glyn O. Phillips Hydrocolloid Research Centre at HBUT, School of Food and Biological Engineering, Hubei University of Technology, Wuhan 430068, China; qdttkl@163.com; 3 Nanjing Institute for Comprehnsive Utilization of Wild Plants, Nanjing, 211111, China

**Keywords:** *indica* rice starch, microwave, storage, chain reassembly, multi-scale structure

## Abstract

The chain reorganization of cooked starch during storage plays an important role in the performance of starchy products such as rice foods. Here, different analytical techniques (such as small-angle X-ray scattering) were used to reveal how microwave cooking influences the chain assembly of cooked *indica* rice starch undergoing storage for 0, 24, or 48 h. While stored, more short-range double helices, long-range crystallites, and nanoscale orders emerged for the microwave-cooked starch than for its conventionally cooked counterpart. For instance, after storage for 24 h, the microwave-cooked starch contained 46.8% of double helices, while its conventionally cooked counterpart possessed 34.3% of double helices. This could be related to the fact that the microwave field caused high-frequency movements of polar groups such as hydroxyls, which strengthened the interactions between starch chains and water molecules and eventually their assembly into double helices, crystallites, and nanoscale orders. This work provides further insights into the chain reassembly of microwave-cooked starch undergoing storage, which is closely related to the quality attributes of starch-based products.

## 1. Introduction

Starch, as a natural polymer, is normally an important food ingredient providing energy for the human body. This polymer contains two types of glucan polymers, including amylose (molar mass: ~10^6^ g/mol) and amylopectin (molar mass: 10^7^–10^8^ g/mol) [1]; these two polymers are organized on different length scales to form a multi-scale structure, involving the starch granule (3–100 μm), growth rings (120–500 nm), blocklets (20–50 nm), lamellae (9–10 nm), long-range crystallites, and short-range helices [1,2]. The structural characteristics of native starch are closely related to its physicochemical properties, such as thermal and texture properties [3,4]. Before consumption, starch often needs a cooking treatment. During cooking, the multi-scale structure of native starch can be disrupted to form a gelatinized starch matrix (mainly sol form), and the viscosity and other rheological features of gelatinized starch can be determined [5,6,7,8]. Then, during cooling and storage, the disordered chains of cooked starch undergo retrogradation (reassembly) to form ordered structures such as crystallites [9,10], and this plays a significant role in affecting the attributes of starch-based food matrices such as mechanical features, sensory features, digestibility, and shelf life [11,12,13,14]. For instance, the chain reassembly of cooked starch into ordered structures tends to increase the firmness and opacity of baked food, thereby reducing consumer acceptance [15,16]. Furthermore, the chain reassembly (retrogradation) of cooked starch can enhance its nutritional significance due to a reduced digestion rate and glycemic response [5,13,17,18,19]. Therefore, to acquire starch-based matrices with improved quality, it is imperative to understand the chain reassembly (into ordered structures) of cooked starch during storage.

There have been reports on the retrogradation of starch cooked using different methods, such as extrusion [20,21,22], autoclaving [23,24,25], and microwave heating [26,27]. Microwave heating, as a rapid heating method, has been widely used in food processing, including baking, cooking, drying, and sterilization [28,29]. Microwave heating involves converting microwave energy absorbed by substances into heat, by inducing polar species (such as water molecules) to rotate [29]. The dielectric features (such as constants and loss factors) of these materials are crucial indicators for evaluating the interaction between the materials and the electric fields [30]. The dielectric properties of starch can depend on the moisture content, system temperature, and starch type [31,32]. It was reported that the dielectric loss factor and dielectric constant of gelatinized starch were higher than those of granular starch [32]. Compared to conduction heating, microwave heating has less impact on the flavor and nutritional quality of food, which is related to the capability of rapid heating [33]. Moreover, microwave processing can change starch crystalline regions and physicochemical properties [26,34,35,36]. For example, an earlier investigation prepared starch suspensions at a 70% water content, which were microwave heated and then stored at 4 °C for 4–72 h; the retrograded starches showed reduced digestibility relative to their native counterparts [26]. Furthermore, microwave treatment can influence the physicochemical properties and digestibility of lotus seed starch, and it was observed that microwave heating was superior to water bath heating in reducing the degree of amylopectin branching [34]. However, while the effects of microwave treatment on the structures and properties of other starches (such as corn and potato) have been reported [26,35,37], there is still limited understanding of how microwave cooking affects the structural features (especially on the nanoscale) of *indica* rice starch undergoing storage. It is known that *indica* rice accounts for more than 80% of rice cultivation worldwide and is an important staple food for more than half of the world population [38,39].

Here, *indica* rice was used as the material for starch isolation. The starch slurries were prepared and cooked using microwave heating and conventional heating. Then, the cooked starch samples were stored for different time periods (0, 24, or 48 h). Different analytical methods were used to inspect the multi-scale structural changes of the starch during the course of storage. The present data are of value for the quality regulation of rice foods subjected to microwave treatment.

## 2. Materials and Methods

### 2.1. Materials

*Indica* rice was obtained from Xiangyang Saiya Rice Co., Ltd. (Xiangyang, China). The starch was extracted according to our previous method [40]. The amylose content of the starch was 14.56 ± 0.39%, and similar amylose contents have been found for *indica* or low-amylose rice grains [4,41]. The amylose content was measured using the method by Sowbhagya and Bhattacharya [42]. All other chemical reagents were of analytical grade.

### 2.2. Microwave Treatment and Storage of Starch

The starch was placed in distilled water to obtain a starch slurry with a starch concentration of 30% (*w*/*v*). The slurry was heated in a microwave oven (MKX-J1A, Qingdao Microwave Creative Technology Co., Ltd., Qingdao, China) at 8 W/g for 3 min. Each of the gelatinized samples was cooled and stored at 4 °C for 24 or 48 h and dried. The freshly cooked starch was freeze dried. The dried samples were crushed and passed through an 80-mesh sieve. The samples were denoted using codes such as “MC-24”, in which MC represents microwave cooking and 24 indicates the storage time. For control studies, the starch slurries (30% *w*/*v*) were heated in boiling water for 30 min to acquire conventionally cooked starches, which were dried and crushed according to the above method. The conventionally cooked starches were denoted using codes such as “C-24”.

### 2.3. Scanning Electron Microscopy (SEM)

The microscopic features of the starch samples were observed at 2000× magnification using a JSM-6390 scanning electron microscope (NTC, Niigata, Japan). The starch samples were placed on conductive tapes and coated with a thin gold film before observations at an acceleration voltage of 15 kV.

### 2.4. Small-Angle X-ray Scattering (SAXS)

The synchrotron SAXS measurements were performed on the BL19U2 BioSAXS beamline at the Shanghai Synchrotron Radiation Facility (Shanghai, China). All the samples were dispersed into distilled water until the moisture reached 80% and equilibrated at room temperature. Then, the samples were filled into 2 mm-thick sample cells, which were covered with Kapton tape. The 2D scattering pattern for each starch was recorded at a wavelength of 1.03 Å using a Pilatus 1 M detector (effective area: 169 mm × 179 mm; pixels: 172 μm × 172 μm). The 1D scattering curves were obtained in a *q* range between 0.015 and 0.5 Å^−1^ from the 2D scattering patterns using the RAW software. The scattering vector *q* was equal to 4πsin*θ*/λ (2*θ*, the scattering angle; λ, the wavelength of the X-ray source).

### 2.5. X-ray Powder Diffraction (XRD)

The X-ray diffractograms of the samples were obtained using an X-ray diffractometer (JDX-10P3A, JEOL, Tokyo, Japan) at a scanning speed of 0.5 °/s and a step size of 0.02°. The starch powders were tested through a 2*θ* angle range of 5° to 40° at 40 kV and 40 mA, with a Cu Kα X-ray source. The relative degree of crystallinity was estimated using the method by Lopez-Rubio, Flanagan, Gilbert, and Gidley [43].

### 2.6. Attenuated Total Reflectance (ATR)-Fourier Transform Infrared (FTIR) Spectroscopy

The FTIR spectra of the samples were obtained on a Nicolet iS50 infrared spectrometer (Thermo Fisher, Waltham, MA, USA) equipped with an ATR accessory from 4000 to 400 cm^−1^. Each spectrum was recorded at a resolution of 4 cm^−1^ for 32 scans against the air as the background. The spectra in the range of 1200 to 800 cm^−1^ were smoothed, baseline corrected, and deconvoluted using the Omnic 8.2 software. The ratio of the peak intensity at 995 cm^−1^ to that at 1022 cm^−1^ (*R*_995/1022_) was used to indicate the relative amount of starch short-range orders.

### 2.7. CP/MAS ^13^C Nuclear Magnetic Resonance (NMR) Spectroscopy

The solid-state ^1^CP/MAS ^3^C NMR spectra were collected at a ^13^C frequency of 100.613 MHz on an Advance AV spectrometer (Bruker, Fällanden, Switzerland), operating at 295.6 K and using a 4 mm broadband double-resonance MAS probe. Approximately 400 mg of the starch powder was placed into a 4 mm-diameter magic-angle spinning sample rotor with a tight push-fitting cap. At least 3000 scans were accumulated for each spectrum with a recycle delay of 2 s. The relative proportions of starch single helices, double helices, and amorphous material were calculated using the PeakFit software (Version 4.12, Systat Software, Inc., Chicago, IL, USA) [44].

### 2.8. Statistical Analysis

The data were expressed as the means ± standard deviations. The statistical analysis was performed using a one-way ANOVA via the 20.0 IBM SPSS software (Chicago, IL, USA). A statistical difference at *p* < 0.05 was considered to be significant.

## 3. Results and Discussion

### 3.1. SEM Analysis for Microscopic Morphology

**Figure 1:**
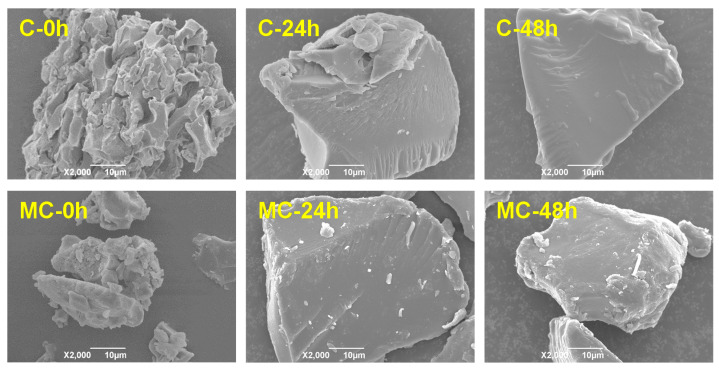
SEM images of conventionally (C) and microwave-cooked (MC) starches with different storage times (0, 24, or 48 h).

Figure 1 shows the SEM micrographs of conventionally or microwave-cooked starches before and after different storage times. The starches after conventional and microwave cooking displayed relatively loose matrices and fragments with a rough surface. This result indicates that the hydrothermal effects during cooking sufficiently disrupted the native starch granules with semicrystalline architectures. While being stored for 24 h, the cooked starches showed irregular shapes with a compact and smooth surface [27,45], similar to that of starches with high temperature–pressure [46] or microwave treatment [47]. Research has consistently reported that autoclaved microwave-treated starches show irregular shapes after storage for 24 h, and the size of starch aggregates exceeded 100 μm [48]. That is, the glucan chains of starch undergo reassembly during storage and increase the bulk density of starch matrices. An increase in the storage time (i.e., 48 h) did not further change the morphological features of the starches, which is consistent with the findings that, after storage, cooked starches acquire a smooth and dense surface [49]. It was noted that there were no significant differences in the morphological changes of the two cooked starches during storage.

### 3.2. SAXS Analysis for Nanostructure

**Figure 2:**
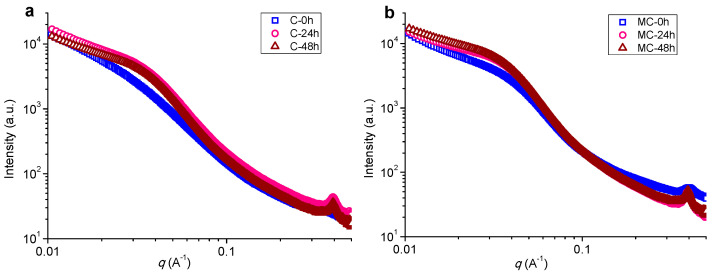
SAXS curves of conventionally (C) (**a**) and microwave-cooked (MC) (**b**) starches with different storage times (0, 24, or 48 h).

The SAXS patterns were used to investigate the nanostructure of cooked *indica* rice starch at the different storage time periods of 0, 24, and 48 h, as shown in Figure 2. When subjected to cooking, the starches (C-0 and MC-0) did not show the typical lamellar peak, suggesting a sufficient disruption of starch semicrystalline lamellae as induced by the hydrothermal effects of cooking [50,51]. The presence of a broad shoulder peak (positioned at ca. 0.03 to 0.04 Å^−1^) indicated the existence of nanoscale (approximately 15–20 nm) starch molecular orders that were randomly distributed in amorphous matrices (namely, nonperiodic amorphous-ordered structures) [52]. The microwave-treated starch (MC-0) had a more prominent shoulder peak than its conventionally cooked counterpart (C-0). This was consistent with the XRD results which showed that, following microwave treatment, the starch was able to reserve more molecular orders such as helices and crystallites.

Storage for 24 h strengthened the shoulder peak of conventionally cooked starch (C-24), but the microwave-cooked starch (MC-24) showed a more intense shoulder peak than C-24. This result again confirms the presence of more nanoscale molecular orders for the microwave-treated starch with storage, and these orders aligned with amorphous regions to construct the nonperiodic structure [53]. The higher number of residual molecular orders after microwave cooking likely plays a role in enhancing the generation of molecular orders after storage. In addition, a 100 inter-helix peak at ca. 0.39 Å^−1^ (about 1.6 nm in size) was observed for the two starches, ascribed to the presence of a B-type crystalline structure [10,54] (also reflected by the XRD technique). A longer storage time (48 h) only led to moderate changes in the scattering intensities at different q values. That is, prolonging the storage time did not prominently increase the content of nanoscale starch molecular orders.

### 3.3. XRD Analysis for Crystalline Structure

**Figure 3:**
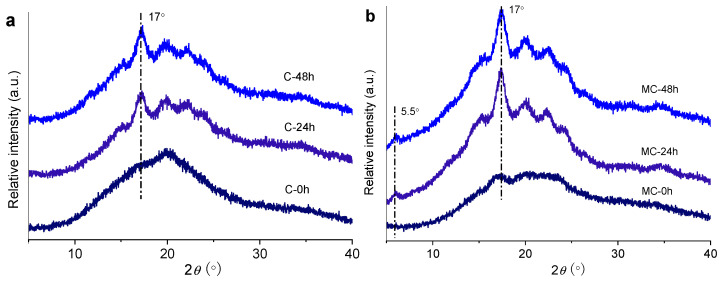
XRD diffractograms of conventionally (C) (**a**) and microwave-cooked (MC) (**b**) starches with different storage times (0, 24, or 48 h).

Double helices are packed laterally into crystalline lattices (long-range ordered structures), which are usually examined by XRD [55]. Figure 3 shows the XRD patterns of conventionally and microwave-cooked starches after different storage times. Before storage, the cooked starches (C-0 and MC-0) displayed a dispersive pattern, ascribed to the disruption of mainly A-type starch crystallites by breaking inter- and intra-molecular hydrogen bonds in crystal cells. The starches that were stored for 24 h showed diffraction peaks at 2*θ* angles of ca. 17°, 20°, 22°, and 24°, corresponding to B+V-type crystallinity [20], which is consistent with a previous report by Ding et al. [45]. This implies that the amorphous chains of cooked starches were assembled into helices, and thus crystallites, during storage [56]. Compared to conventionally cooked starch (C-24), the microwave-cooked starch (MC-24) showed stronger diffraction peaks, especially at 17°, with an additional peak at approximately 5.5°. Microwave heating led to a residue with more starch orders than the conventionally cooked starch (as indicated by the stronger diffraction peaks for MC-0 than for C-0), which served as crystal seeds to facilitate the crystallization of starch chains induced by storage. A further increase in the storage time slightly affected the diffraction peaks for the two cooked starches, and the positions of the diffraction peaks did not change.

### 3.4. ATR-FTIR Analysis for Short-Range Orders

**Figure 4:**
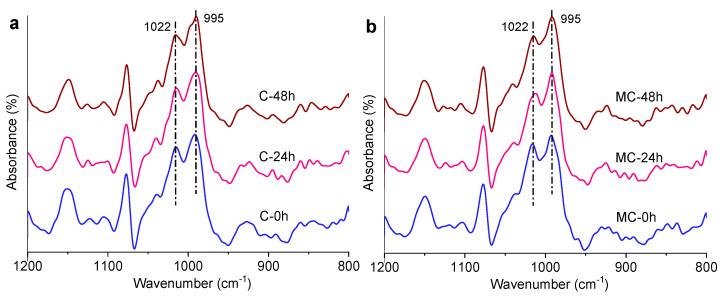

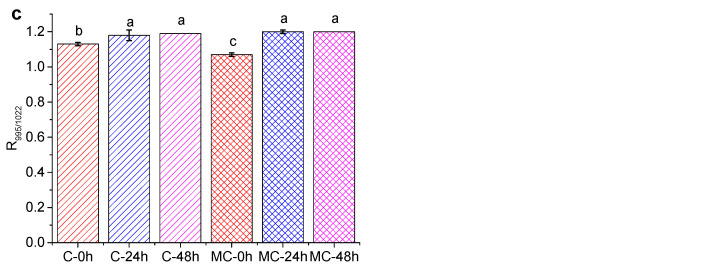
ATR-FTIR patterns: (**a**) conventional cooking; (**b**) microwave cooking; and (**c**) the intensity ratio (*R*_995/1022_) values of conventionally (C) and microwave-cooked (MC) starches with different storage times (0, 24, or 48 h). The different lowercase letters above the data bars in (**c**) indicate a significant difference at *p* < 0.05.

Two adjacent branches of amylopectin could form double helices (short-range ordered structures) [55]. The ATR-FTIR technique is typically used to evaluate the short-range order degree of starch [10]. The selected spectra in the range of 1200 to 800 cm^-1^ for the starch samples are shown in Figure 4. The band at about 1022 cm^−1^ is associated with the relative content of amorphous starch materials, and the band at ca. 995 cm^−1^ reflects the content of short-range orders such as starch helices [57,58]. The two peaks are highlighted using dashed lines in Figure 4a,b. The intensity ratio (*R*_995/1022_) of the band at 995 cm^−1^ to that at 1022 cm^−1^ can be used to indicate the content of short-range orders, and the results are presented in Figure 4c. It was revealed that the conventionally cooked starch (C-0) had an *R*_995/1022_ close to that of the starch subjected to microwave treatment (MC-0). While storage for 24 h resulted in a comparable *R*_995/1022_ for the two types of cooked starches, a longer storage time did not further change the *R*_995/1022_ value. It has been found that the short-range order absorbance ratio of banana starch reaches a plateau after storage for 11 h [59]. Considering the SAXS and XRS results, microwave cooking followed by storage produced more nanoscale orders (packed in nonperiodic structures) and long-range scale orders (crystallites), rather than short-range scale orders (helices). Furthermore, increasing the storage time only produced a similar amount of short-range ordered structures for the starches with different cooking manners.

### 3.5. ^13^C NMR Analysis for Short-Range Orders

**Figure 5:**
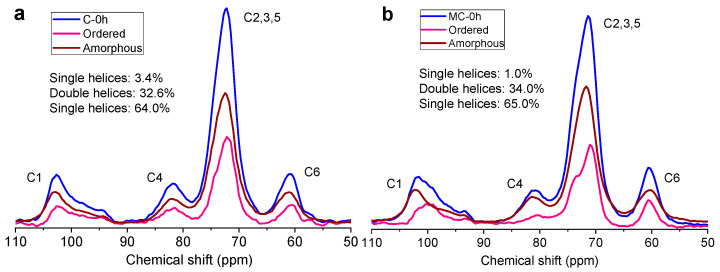

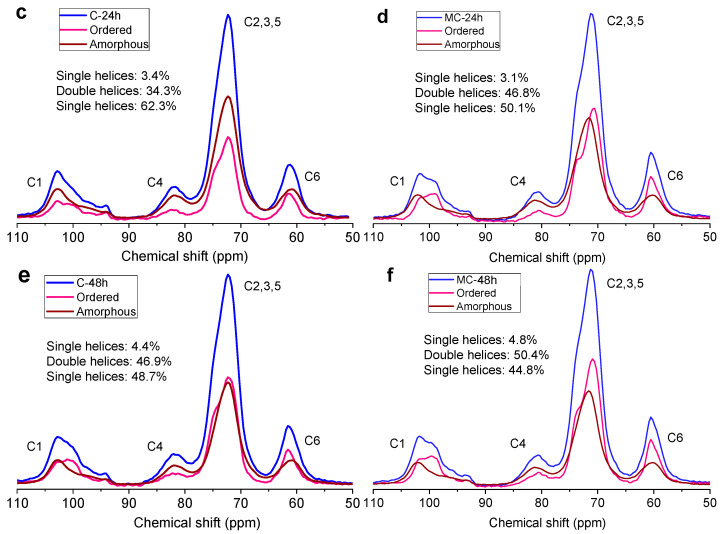
^13^C CP/MAS NMR curves of *indica* rice starch after conventional cooking (C) (**a**,**c**,**e**) and microwave cooking (MC) (**b**,**d**,**f**) at different storage times (0, 24, or 48 h).

^13^C CP/MAS NMR has been applied to estimate the relative proportions of single- and double-helical components of starch [40,60]. The ^13^C CP/MAS NMR profiles of the starches and their ordered sub-profiles are presented in Figure 5, in which the starch samples exhibited C1 (94–105 ppm), C2, 3, and 5 (68–78 ppm), C4 (80–84 ppm), and C6 (58–65 ppm) signals. The C1 and C4 peaks were related to ordered and amorphous starch components, respectively [61]. In Figure 5, the total content of single and double helices in C-0 was about 36%, being similar to that in MC-0 (approximately 35%). After storage for 24 h, the microwave-cooked starch (MC-24) contained more double helices than the conventionally cooked starch (C-24), followed by a similar content of single helices (ca. 3%). The present results are unlike those of retrograded pea starch that contained 1.43% to 2.30% of single helices and 57.5% to 58.25% of double helices [62]. Furthermore, the ordered sub-spectrum of MC-24 showed an unresolved doublet peak in the C1 range (Figure 5d) due to the presence of B-type crystallites with hexagonal crystal units [40]. The single helices were associated with V-type starch crystallites. While a longer storage time increased the content of short-range orders, the starch following microwave cooking also presented a higher content of single/double helices [62]. Hence, compared to the FTIR technique, the NMR technique was more sensitive to the starches’ helical structures and more clearly presented the changes within such ordered structures.

### 3.6. Discussion on How Microwave Cooking Induced More Starch Orders after Storage

The molecular chains of cooked starches can reassemble into ordered structures during storage, largely affecting the properties of starch matrices such as hardness and digestibility. This chain reassembly event involves several key steps: (i) amorphous chains are organized into single helices (via hydrophobic force) and double helices (via hydrogen bonds); (ii) the single helices aggregate into V-type crystallites, and the double helices align with water molecules via hydrogen bonds to construct monoclinic crystal cells (A-type crystallites) and hexagonal crystal cells (B-type crystallites); (iii) the helical and crystalline components further aggregate to form nanoscale ordered regions, and these regions randomly distribute in amorphous regions [2,13,40]. The starch chain reassembly (retrogradation) could be affected by a series of factors, such as water content, storage temperature, and additives (salts, lipids, proteins, etc.) [13].

The theory here and the above results could enable an understanding of how microwave cooking followed by storage induces more nanoscale and short-range orders in starch. Specifically, the two cooking methods sufficiently disrupted the semicrystalline architecture of native starch, as indicated by the residues with similar amounts of short-range scale orders in cooked starches. Unlike conventional (conductive) cooking, the electromagnetic effects of microwave cooking could induce high-frequency movements of polar species, such as hydroxyl groups of starch chains and water molecules. This fact probably increased the opportunities for starch chain–water interactions. Consequently, microwave cooking followed by storage not only substantially enhanced the formation of short-range orders (chain self-assembly into helices) during storage but also facilitated the construction of long-range crystallites (containing helices with water molecules) and then nanoscale orders (aggregation of short- and long-range orders). Furthermore, the greater number of long-range scale and nanoscale orders in microwave-cooked starch could serve as seeds to promote the formation of ordered structures.

## 4. Conclusions

This work confirms that microwave-cooked starch following storage possessed more short-range, long-range, and nanoscale ordered structures, relative to the conventionally cooked starch. While the two cooking manners reserved a similar number of short-range helices, the electromagnetic effects of microwave heating likely induced the high-frequency movements of polar groups (such as hydroxyls) and thus enhanced the interactions between starch chains and water molecules. This was preferable for the chain alignment into short-range double helices, long-range crystallites (aggregated from helices and water molecules), and nanoscale orders (aggregated from helices and crystallites). Consistently, storage for 24 h led to more double helices (46.8%), B+V crystallites, and nanoscale orders for the microwave-cooked starch than for its conventionally treated counterpart. A further increase in the storage time slightly affected the ordered structural features on different scales. This work enables a better understanding of the chain reassembly of microwave-cooked starch during storage, which is of value for the design of starch-based products (such as ingredients) with tailored qualities such as digestibility.

## Data Availability

Data is contained within the article.
